# Improving research and development of wearable assistive and rehabilitation technologies: a systematic review on diversity factors

**DOI:** 10.1186/s12984-025-01562-7

**Published:** 2025-02-21

**Authors:** Mariya Lorke, Thekla Stefanou

**Affiliations:** Faculty of Engineering and Mathematics, University of Applied Sciences and Arts (HSBI), Bielefeld, 33619 Germany

**Keywords:** Diversity, Assistive technology, Rehabilitation technology, Wearable technology, Exoskeleton, R&D, Intersectionality, User experience, Ethical considerations

## Abstract

This paper analyses diversity and intersectionality aspects in the R&D of wearable assistive and rehabilitation technologies (WEARTechs). We advocate for inclusive, innovative research that we hope will help bridge the gap between laboratories and the real world and reduce disparities in healthcare and technology development. We performed a systematic literature review of the intersections between assistive technologies and diversity and conducted a thematic analysis of the diversity factors identified in the literature. In addition, we carried out a supplementary literature search on WEARTechs to discover which, if any, diversity aspects are currently being reported on. Our findings indicate that diversity has not been addressed in the field of WEARTechs. There is not sufficient knowledge to determine, which diversity-related aspects researchers must consider when evaluating the performance of any specific WEARTech device. Nor about how these can be properly addressed in the R&D process. We, therefore, provide actionable recommendations on how to integrate diversity-relevant aspects at different R&D stages. We hope that our review will help scientists rethink and reformulate approaches to the R&D of WEARTechs and build the way towards more inclusive solutions. It is our belief that this will spark innovation and enhance discovery potential in the field.

**Supplementary Information:**

The online version contains supplementary material available at 10.1186/s12984-025-01562-7.

## Introduction

The first relevant publication on rehabilitation and assistive technologies found in SCOPUS is “Rehabilitation research: a fertile field for new technology” by Carlos Vallbona, published in 1969. Scientists and engineers have, since, achieved great innovations in the field, providing new opportunities for impaired individuals in rehabilitation and everyday life. This exciting field at the interface of humans and machines, opens up not only space for technical endeavour and innovative design thinking but also for critical reflection on technology and society, as well as how research and development in the field is done. To address diversity in this area of research, we performed a systematic review on diversity aspects during the research and development (R&D) of wearable assistive and rehabilitation technology (WEARTech). We define a WEARTech as any wearable product or technological development that is part of a wearable device, hardware or software, designed for or used with the purpose of enhancing at the physical functioning of an individual with movement deficiencies.

As the field of WEARTechs is an evolving field characterised by ongoing change, representative data on their use are still lacking and terminology used varies across studies and disciplines. In the reports provided by the World Health Organization (WHO), assistive technology (AT) is a broader term for technologies which include WEARTechs, prostheses and more general assistive and rehabilitation technologies. A global epidemiological perspective emphasises the high relevance of developments in the field of ATs: more than 2.5 billion people around the world would benefit from at least one assistive product and this number is expected to rise to over 3.5 billion by 2050 [1].

Concerns have been expressed over device accessibility, usability and potential abandonment by users [2]. In a systematic review on robotics in rehabilitation, Laparidou et al. determined that technological challenges were one of the major barriers to device adoption [3, 4]. Even after accessibility and adoption hurdles have been overcome, the abandonment rates in users appear to be significant [5, 6], in particular within a year, with evidence that the figures are higher amongst women (39%) than men (23%) [6]. The impact of abandonment can be high; fall rates of individuals that abandoned a mobility device were found to be significantly higher than those that continued its use [7]. In [6] the most common factors for abandonment by users were found to be “just as or more functional without it” and “more functional without it”, while an older study states “lack of consideration of user opinion in selection, easy device procurement, poor device performance, and change in user needs or priorities” [5].

Still, little is known about the expectations potential users and their significant others have of AT [8] or how ATs perform across the diverse target user population. This is surprising as we know that body morphology differs across genders and ages; some difference are evident, such as hip width and chest size, but others such as pressure pain thresholds, that would affect comfort of attachment points, are not. Pressure pain threshold studies comparing genders have a prevalent theory that female and male bodies’ response to pressure is different depending on location [9, 10].

The purpose of AT, according to the WHO, is to enable people to live healthy, productive, independent and dignified lives, and participate in education, the labour market and civic life independent of their gender, age or other diversity characteristics. It should enable and promote inclusion and participation of individuals with disability, ageing populations, and people with non-communicable diseases; maintaining or improving an “individual’s functioning and independence, thereby promoting their well-being” [1, 11]. The concepts of well-being and good life are subjective and mirror certain cultural norms and values. It is well known that such norms and values are also inscribed into technology [12]. On the one hand, these are related to the socially acceptable and dominating beliefs and virtues related to the body, technology, individual health and social participation [13]. On the other hand, they reveal the implicit assumptions of developers and manufacturers about users of this technology and their anticipated needs [12]. This cultural and social dimension of technology opens up space for ethical and moral reasoning on possible harms that potentially non-ethical technology introduction may cause on users and society in general. For example, a gender divide in technoscience has been the subject of critical research [14–16], whereby a privilege gap between individuals with different diversity characteristics in STEM-research was observed [17]. Furthermore, exclusion and reproduction of discrimination of marginalised and under-represented groups through technology were increasingly thematised [18, 19]. These critical dimensions are also valid in the field of AT and offer opportunities for improvement. This issue was recently recognised by the international journal ‘Assistive Technology’, whereby a call for explicitly addressing diversity in R&D was made [20].

Two main perspectives—engineering and socio-anthropological—informed the study design and hypothesis formation. WEARTechs work closely with the human body; at the physical interaction and sensory levels. This means that they have a small margin for error in fit and adaptability to the human body, and appropriate torque transfer is important. A review on exoskeletons developed specifically for nurses (the majority of whom female), concludes the study with a list of “the most important characteristics desirable for an exoskeleton for nurses”. The first bullet point is “design with focus on women’s body shape” [21]. This is one example that illustrates the impact the neglecting diversity factors during the design process has on users. Additionally, some of the most common actuation strategies of WEARTechs include the user in the control loop [22]; human-in-the-loop strategies can be very effective but also need to take into account the fact that ‘humans’ are not a monolith. Failing to perform well in any of these aspects could not only lead to the abandonment of the device, but also injury. We do know that electromyography (EMG) signals are affected by skin properties that have been found to differ across skin colours and thus racial backgrounds [23, 24]. Do differences in body morphology and biosignals, such as EMG, used for inferring motion intent, affect the performance and usability of systems that use them at the human-WEARTechs interface?

To our knowledge, no systematic literature review on the intersectionality between assistive technologies and diversity has been carried out so far; a broad bottom-up approach towards operationalising the concept was needed. Thereby, diversity-related phenomena in the field of ATs were collected and analysed in order to set the foundation for a framework for approaching diversity in WEARTechs. In this article, diversity is understood as the plurality of perspectives and diversity characteristics of groups and individuals [25, 26]. Diversity is not understood as a way of categorising otherness, but rather, as an ‘anthropology of the otherwise’ [27]; where a variety of lifeworlds (subjectively experienced everyday life of individuals) and perspectives in the context of AT R&D and use become visible. Instead of solely focusing on the terms and characteristics of diversity, the concept of diversity employed in this article looks at the standards to which the world is observed and enacted by society’s participants so that it can be seen and understood as diverse ([28], p.16). As WEARTechs and ATs in general are meant to serve individuals and not entire groups, an intersectional approach towards technology development is needed. The intersectional approach does not address single diversity characteristics, but their interconnectedness. Gender, race, class, sexual orientation, ethnicity, disability and other characteristics are examined in terms of their interdependence and interaction in individuals [29].

The overarching research question of this article is on the role diversity factors play in WEARTechs R&D and on their impact on device performance and usability. Contemplating the various facets of this research question we formulated hypotheses (Table 1) based primarily on the background literature presented, and identified aspects of the WEARTech field that are potentially being influenced as a result.

**Table 1:** The hypetheses formulated on diversity aspects in WERTech research and development

No.	Hypotheses	Aspects
H1	Diversity and intersectionality aspects have not been adequately considered in the field of WEARTechs.	grants, research proposals product development
H2	There is a lack of diversity in the field of WEARTechs R&D.	researcher groups, R&D practices
H3	Participant pools in WEARTechs R&D are not representative of the target population.	reported results are misrepresenting performance
H4	The technological developments and performance in the field of WEARTechs mirror the lack of diversity considerations during R&D and lack of diversity in R&D.	physical interface & body morphology, biosignals & technology adaptability
H5	Each stage in R&D has unique nuances and thus different diversity considerations are of importance.	user involvement diversity characteristics

The aim of WEARTechs is to meet the physical needs of AT users. Researchers, engineers and policy makers need to **(i)** learn and understand more about the differences across users and the effects the use of each AT has in an individual’s life and everyday experiences, and **(ii)** incorporate that to the design, development and introduction of WEARTechs into the users’ lives.

## Methods

An appropriate methodology to address the research question defined above and synthesise the best available research is a systematic review. On the one hand, this method allows for systematising evidence on diversity factors in various studies on AT, even though these factors were not recognised and named as such by the authors of the included studies themselves. On the other hand, we believe that a systematic review in the field may support practice, R&D and demonstrate the need for additional funding in this research field. Although our focus is on WEARTechs, given the novelty of the field, we broadened our search to general ATs. We carried out a systematic literature review (Phase I) to identify the variety of aspects as well as to find out different areas of intersection between diversity and technology in individual lifeworlds. Our methodological approach is based on established methods and recommendations on conducting systematic reviews (PRISMA template [30]) and combines characteristics of systematic reviews in engineering and social sciences. In order to better contextualise the findings of the systematic review we decided to conduct a supplementary literature search specifically in the field of WEARTechs (Phase II), without explicitly including diversity factors in the search algorithm. The goal of this supplementary research was to look at how latest R&D is actually being carried out and reported on in terms of diversity aspects.

### Phase I: systematic review

The four databases used -SCOPUS, PubMed, Web of Science (core collection) and Science Direct- were chosen by consulting published literature reviews in engineering and social sciences journals and via a preliminary testing of search algorithms. We initially performed single limited searches testing search terms, which derived from the core concepts (assistive or rehabilitation technology and diversity). In this testing phase, we analysed relevant key words and put together a list of search terms. These were compiled to form the initial search algorithm, which was then adapted to each database, according to its capabilities and restrictions, to maximize the relevance of the search results (Table 2)^1^. Additionally, for each database search the available filters were used to ensure the language and accessibility inclusion criteria were satisfied. Where available, we filtered out articles of irrelevant subject areas and carefully adjusted the filter to exclude medical-focused publications, that were procured due to the use of the “disability” term, without sacrificing the breadth of relevant results.

**Table 2:** Overview of the query algorithm used at each databases and returned results

Databases	Terms / Search formula	Results
SCOPUS	(((rehabilitation OR assistive OR rehabilitative OR assistance OR disability) AND (device OR technology)) OR	227
	exoskeleton) AND (( gender OR sex OR cultural OR race OR racial ) AND (intersectionality OR diversity OR	
	Inclusivity OR inclusive OR intersectional OR identity))	
	AND limit subject area to: social sciences, computer science, engineering	
	AND limit the languange to: English	
	AND limit the document type to: Article, Book chapter, Review, Short survey	
PubMed	*Title/Abstract*: (((rehabilitation OR assistive OR rehabilitative OR assistance OR disability) AND (device OR	127
	technology)) OR (exoskeleton OR exosuit)) AND (gender OR sex OR cultural OR race OR racial OR	
	intersectionality OR diversity OR inclusivity OR inclusive OR intersectional OR identity)	
	AND limit the language to: English	
Web Of Science ( core collection )	(($^{\textrm{1}}$TI=((((rehabilit* OR assist*) AND (device OR technolog*)) OR exoskeleton) AND ((gender OR sex OR	226
	cultural OR race OR racial) AND (intersection* OR divers* OR inclusivi* OR identity)))) OR	
	$^{\textrm{2}}$AB=((((rehabilit* OR assist*) AND (device OR technolog*)) OR exoskeleton) AND ((gender OR sex OR	
	cultural OR race OR racial) AND (intersection* OR divers* OR inclusivi* OR identity))) OR	
	$^{\textrm{3}}$AK=((((rehabilit* OR assist*) AND (device OR technolog*)) OR exoskeleton) AND ((gender OR sex OR	
	Cultural OR race OR racial) AND (intersection* OR divers* OR inclusivi* OR identity))))	
	AND Document types: Article, Review Article, Book Chapter, Editorial Material	
Science Direct	*Title, abstract or author-specified keywords: *(((assistive OR rehabilitation) AND (device OR technology)) OR	184
	exoskeleton) AND (intersectionality OR diversity OR identity)	
	AND Article type: Review articles, Research articles, Book chapters, Case Reports, Discussion	
	AND Subject areas: Engineering, Social Sciences	

$$^{1}$$TI = title

$$^{2}$$AB = abstract

$^{3}$AK = author keywords

The inclusion criteria dictated that articles had to: be written in English and address at least one diversity aspect in the context of ATs. After screening the resulting literature, the researchers defined the following exclusion criteria:

(i) articles that address solely the introduction and use of exoskeletons in the military field and factory-based work,
(ii) articles that present or evaluate instruments for AT selection or instruments for decision making on AT,
(iii) articles that refer to ATs that address types of disability other than mobility (e.g. visual or hearing impairments, cognitive, etc.).

Studies were considered eligible if they addressed at least one diversity aspect in the field of ATs, offered empirical findings that relate to aspects of diversity or demonstrate neglect of diversity and/or provide theoretical anchoring of concepts related to diversity in the field of engineering and ATs.

**Fig. 1:**
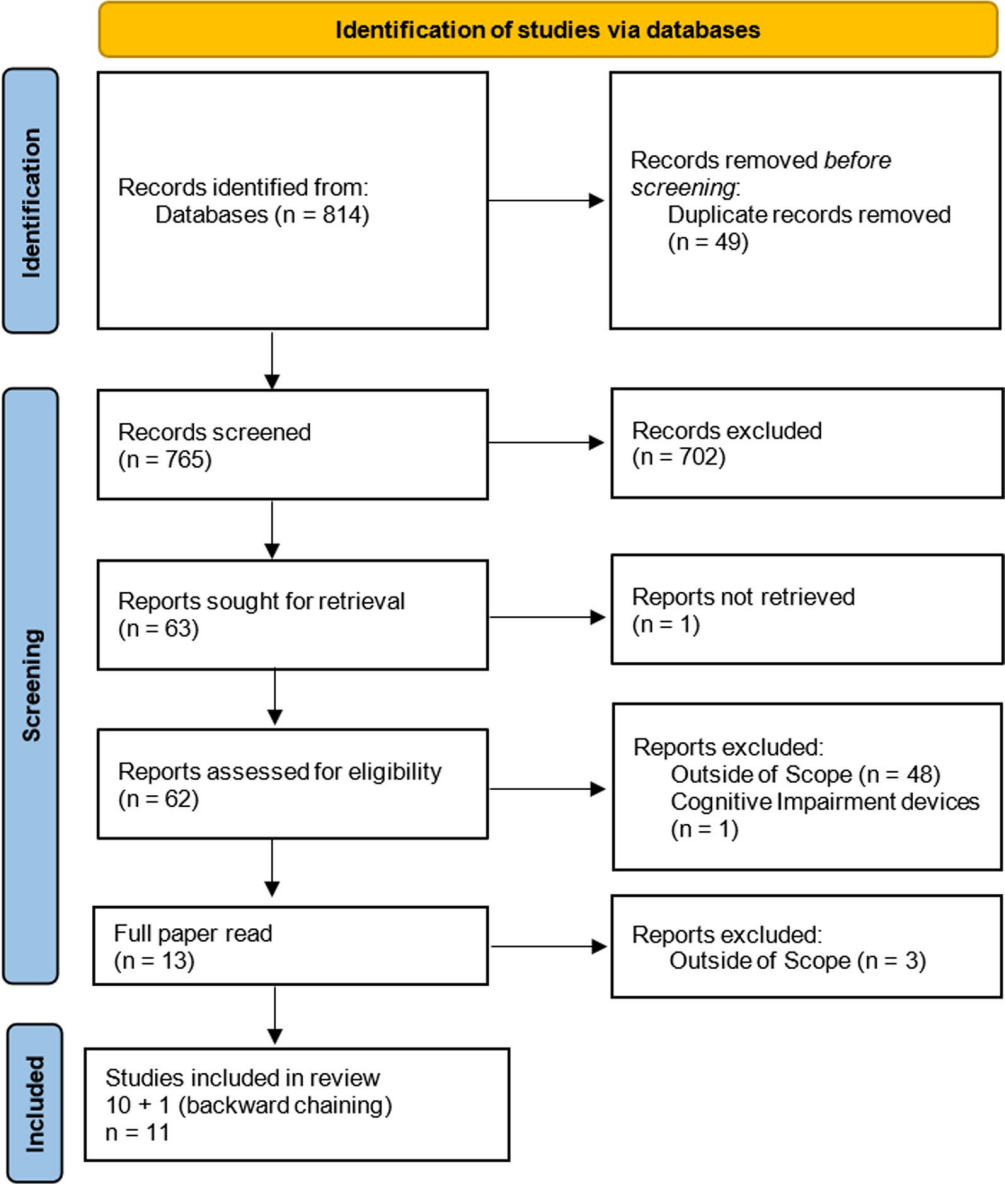
The *PRISMA* flowchart presents the results of the selection process, tracking the number of articles through each scanning and review step. Duplicate records were removed in the *Identification* stage prior to screening . In the *Screening* stage the titles and keywords were screened to determine relevance and, following that, the eligibility criteria were applied based on the abstract. This was followed by full text reviews and backward chaining for additional relevant articles

The search covered journal publications, conference proceedings, preprints, reports, and book chapters written in English. In total 814 articles were retrieved through the initial search conducted in February 2024. The final results were exported to a literature management program, citavi (Swiss Academic Software, Switzerland), and duplicates were removed, in the *Identification* phase (Fig. 1), resulting in 765 unique articles. The screening process is documented in the PRISMA flowchart [30] presented in Fig. 1 and detailed in the Literature search.

The retrieved literature was summarised, focusing on the diversity-related aspects of ATs and a thematic analysis was performed. We analysed the diversity aspects found in: (1) technologies, (2) users, (3) use/usage and (4) perspectives. As the concept of diversity is multi-faceted, it was not possible to draw a clear line between the four categories and it should be noted that some of the aspects discussed may overlap. The relevance of these findings (research, development and use of AT) to the field of WEARTechs was then discussed in order to identify implications for research, development and use.

### Phase II: supplementary search on WEARTech state-of-the-art

The conducted systematic literature research (*Phase I*) addressed articles in the field of ATs in general, providing insights into relevant diversity aspects. Nevertheless, the retrieved literature could not provide answer of the question how diversity-sensitive the field of WEARTechs actually is. In order to bridge that gap, address the questions we set to answer and provide further context to our systematic review, a supplementary literature search on the state-of-the-art in WEARTech, was carried out. The questions we set out to answer addressed diversity within participant pools in WEARTech studies through the different stages of assistive and rehabilitation technology development (*H3*) and the diversity in research groups (*H2*). The keyword syntheses shown in Table 3 were used to perform the search in the *Web of Science* and *SCOPUS* databases. The search was performed on February 25th 2024 and was limited to the publications of the last 5 years written in English. No other filters were used to refine the search as the results were sorted by ‘relevance’ and the top 100 from each database were sampled. The publications were then all screened using the abstract and the ones that used previously published participant data or focused solely on simulations were excluded. After the screening a total of *57* papers were left. The information extracted from the chosen papers included: the type of device, what participant information was recorded, the stage in the R&D process, the number of participants and their genders (where feasible), the number of authors and their genders, and the country in which the institutions that supported the research are situated. The results of this literature search are provided in the supplementary materials and are solely used to better contextualise the central findings of the systematic review (conducted in Phase I).

**Table 3:** Phase II: WEARTechs literature research

Database	Keyword synthesis
Web of Science	$^{\textrm{4}}$TI=(((rehabilit* OR assist*) AND (device OR technolog*)) OR exoskeleton OR ((active OR powered) AND orthos*)))
SCOPUS	TITLE-ABS-KEY(((rehabilit* OR assist*) AND (device OR technolog*)) OR exoskeleton OR ((active OR powered) AND orthos*))

$$^{4}$$TI = title

## Results and discussion

### Literature search

Following the systematic screening process of the 765 unique articles found during the literature search (Fig. 1), sequential elimination was used to retrieve the relevant articles for this review on the diversity factors explored in WEARTechs R&D. To start with, the body of literature found was split in half and each half was assigned to one of the two authors for the first screening; judgement on the relevance of each article was based on the article title and keywords. This was then cross-checked by the second author and a final decision on eligibility was made. In cases of diverting judgments the articles were included. Only 63 articles were left after this first *Screening* step. Of those, one publication could not be retrieved (broken link) and the abstract was not available. In the next step, we performed an eligibility assessment based on the abstract or summary of each of the 62 retrieved citations (third step of the PRISMA Screening stage); this is when the exclusion criteria were also applied. All publications were screened by both authors and diverting views on the eligibility of any articles were resolved through discussion. The rejection of 49 papers left us with 13 articles. These were scanned for manual records and their references were proofed for eligibility, ie. backward citation chaining. The last step in the *Screening* stage consisted of two parts. The first was the full text reviews of the 13 remaining articles, where 3 further articles were excluded due to insufficient relevance, resulting in 10 papers. The second part was the identification of relevant articles through backward chaining and their full paper review. Five articles were identified using backward chaining and, of those, one was added to the body of literature. This brought the total of the articles that were deemed eligible for data abstraction to 11. The data extracted from these articles are presented in Table 4 (an extended version of which can be found in the supplementary materials). As none of the included articles explicitly examined or measured one or more diversity factors in WEARTechs, the appraisal of the studies’ general quality in terms of the methodology they used was not relevant. For this reason the appraisal of the articles took place solely on the way diversity factors were addressed. Both authors appraised all articles independently and negotiated on their findings. The consolidated appraisal (including information on potential biases) is available in the supplementary materials.

**Table 4:** Tabulation of the extracted data from the resulting literature of the systematic review performed on the intersectionality between assistive technologies and diversity

No.	Title	Type	Research Design	Results	Diversity aspects
#1 [31]	The influence of social context on the perception of assistive technology: using a semantic differential scale to compare young adults’ views from the United Kingdom and Pakistan	original research	Mixed-methods research on perceptions of assistive technologies in two countries, reflecting on differences between individualist and collectivist societies, whereby an online questionnaire was applied. The questionnaire contained three sections: (1) participant socioeconomic profile, (2) Semantic Differential (SD) scale to characterise participants’ opinion using three factorial categories—evaluation, potency and activity, (3) opinions about users.	Overall, no statistically significant difference between the two cultural groups (UK and Pakistan) was found. Perception of usefulness/ease-of-use differed among participants of both groups, while perception of usability of ATs (wheelchair) was the same. For young members of society, those with prior experience of interacting with wheelchairs, had a more ‘positive perception’ of them, compared to those without. In general, UK group responses were skewed towards a more negative view of disability compared to the Pakistan group. However, it is important to note that, a lot less UK respondents had either used or were carers for users of wheelchairs compared to Pakistan (104 vs. 307). Both groups used negative adjectives to describe the user and the authors associated these results to the existence of stigma in both societies; stigma associated with this AT product was greater in the UK.	Culture, ethnicity, society, disability, gender, profession
#2 [32]	How can ISO 13482:2014 account for the ethical and social considerations of robotic exoskeletons?	review article	A systematic review of personal care robot/exo/“Physical Assistants” regulatory standards was performed, whereby shortcomings and gaps of relevant ISOs were identified. This analysis may help facilitating the introduction of robots in healthcare.	The results of the analysis showed that ISO 13482:2014 failed to sufficiently and comprehensively address safety, overlooking critical legal, ethical, and social considerations, which can compromise safety. The following areas of improvement were identified: scope, definitions, hazards, safety design measures, verification and validation etc. The study emphasised the “shared responsibility” among all stakeholders within the personal care robot ecosystem in ensuring “the comprehensive enhancement of safety and functionality across all facets of personal care robot technology”.	Body morphology, gender, disability
#3 [33]	Accounting for diversity in robot design, testbeds, and safety standardisation	original research	The study explores the impact of overlooking gender and sex considerations in robot design. A mixed methods study design was employed, consisting of quantitative research monitoring heart rate (stress), kinematics (functionality, protective stops, graceful collapsing and stability), and qualitative research by means of pre- and post-test questionnaires (on fear of falling, acceptability, perception and stress). The study is based on two experiments: 1) focused on gaining insights on the safety-related aspects of exoskeletons from a diverse group of subjects; 2) focused on gaining more in-depth insights into the exoskeleton safety-related aspects.	Unique characteristics of female, elderly and disabled users that are safety-critical were identified: morphological differences between m/f (m/f pelvises, breasts); differences between men and women regarding control over one’s own body; women experience the exo as heavier than men do; elderly users, users who have a medical condition, or those who walk again after having been in a wheelchair for an extended period of time, might find it more challenging to adapt to an exoskeleton, and this may lead to higher safety risks. The study concludes that the standards should include clauses on safety requirements and hazard lists for different user categories. Devices should state which users can safely use it. The authors recommend taking a more holistic, user-centred approach toward robot safety, taking into account user’s perception on risk and safety associated with devise-use. Device design & testbed design should: consider different users’ needs, ensure ’design justice’, comply with minimum safeguard baseline and personalise on top.	Gender, disability, age
#4 [34]	The shaping of individual meanings assigned to assistive technology: a review of personal factors	review article	Comprehensive review on ’individualised’ meanings assigned to assistive technology and how these influence their integration into daily activities. Review of evidence on AT use and non-use by: (1) older adults; (2) persons with acquired disabilities; (3) persons with congenital disabilities; and (4) persons with functional limitations due to progressive diseases, for the purpose of identifying the psychosocial, cultural and adaptation factors that shape individual meanings ascribed to AT.	The study demonstrates how the psychosocial and cultural issues (summarised as personal factors) influence the shaping of individualised meanings assigned to AT. The synthesis of evidence on older adults does not contain information on AT related to mobility but shows that AT meanings of elderly individuals are shaped by desire for independence, control and cost savings. The formulation of meanings ascribed to AT, by persons with acquired disabilities, is influenced by personal factors including: (1) a sense of control; (2) level of desired independence relative to other values; (3) mechanical performance; (4) changes in physical and health status; (5) preservation of preferred self-image; (6) functionally defined time-frames; and (7) promotion of enjoyable activity participation. The evidence suggests that persons with congenital disabilities formulate meanings ascribed to AT based on personal factors that include: (1) tolerance for communication breakdowns; (2) desire to belong to a peer group; and (3) sense of conversation control. Overall, the successful integration of AT into daily activities requires potential device users to explore: (1) the meanings they assign to devices; (2) their expectations; (3) the anticipated social costs; and (4) ways to come to terms with disability as one of, but not the defining, feature of oneself.	Age, gender, disability, psychosocial factors, culture
#5 [35]	Living with assistive robotics: exploring the everyday use of exoskeleton for persons with spinal cord injury	original research (case study)	The study applies a qualitative research design based on ethnographic fieldwork (incl. interviews and cultural probes).	This case study provided insight into the lived reality of using the ReWalk exoskeleton. The results showed that the appropriation and integration of AT within the patients’ everyday lives required a social and collaborative effort, which continued into use. The decisions to utilise the technology (or not) was closely tied to physical, social, cultural, environmental, and psychological factors. Four themes emerged: (1) Meaning of mobility- it represents more than functional ability; its present socio-cultural meaning is associated with an individual’s self-identity and life priorities. (2) Accomplishing body technique-integration with the body requires a long process of skill acquisition and re-embodiment. (3) Adaptation and adjustment in use-successful use of the technology was characterised by ongoing adjustment and adaptation of the technology and ways of using it. (4) Human element-introduction and sustained use of the exoskeleton demand a social and collaborative effort across the user’s professional and lay resources. Possible practical implications are: developers (and suppliers) need to work more closely with patients, developers should be aware that the “mechanical workings of the device demand new body techniques and the impact that cognitive load has on its acceptance, technology should be built with an understanding of the individual’s life.	Diversity in lifeworlds and living experiences
#6 [36]	The Assistive Technology Passport: a resource for enhancing capabilities as a result of better access to assistive technology	original research	The study explores factors that affect access to AT. A qualitative research is performed using capability approach, lived experiences and Interpretive Phenomenological Analysis. Data was collected via a semi-structured in-depth interviews.	A consistent focus across participants was the human agency to live a valued life of their choice and the recognition of human diversity as a significant factor for the well-being of the person and the wider society. The participants further explored the potential for developing an AT ’passport’ as a capability-enhancing resource for access to AT. AT personnel often lack understanding and clear guidelines on the AT provision pathways. Subsequently, user involvement in assessing and providing an appropriate AT is limited, resulting in the provision substandard products and leading to abandonment.	Values, lived experiences and freedom of choice
#7 [37]	Sensing technologies, digital inclusion, and disability diversity	original research	A qualitative research design was applied in this study. Data was collected via three workshops with community leaders (n=6), Disability advocacy focus group (n=11) and self and peer advocates (n=10). Themes discussed included: (a) access to technologies at home; (b) use of technologies to enhance everyday life living with an impairment; (c) prominence and meaning of technology in their daily life; and (d) activity-focused technology use (work, recreation, mobility/transport, shopping, personal networks, and service use).	The sensory capacities of consumer devices-fingerprint scanning, and voice and touch sensors-are significant for privacy and accessibility. Participants discussed sensory technology failures to capture accents, gestures, movements, or voice commands expressed in a non-normative way. Participants complained about lack of access to materials in plain English about sensory technologies, an issue that is particularly challenging for those with intellectual impairments or those whose first language is not English. Financial hardship was found to be the largest barrier to digital inclusion. Many participants noted the, often, invisible work is undertaken by volunteers, family, and carers who make the technologically accessible through acts of interpretation and support. The study findings illustrate that the design of sensing technologies often assumes a medical model of disability rather than seeing access as a collective responsibility, distributed throughout the technological service provision supply chain. Such normative assumptions instead require individuals with disabilities to adapt or avoid them.	Language, cultural background, disability, minority status, socioeconomic status
#8 [38]	An exploratory study analysing demographics and opinions of assistive technology professionals within the complex rehab technology industry	original research	A quantitative research design using a survey on AT professionals was applied. The survey data was analysed by means of descriptive statistics.	This exploratory study investigated the demographics of Assistive Technology Professionals (ATPs) regarding age, education, certifications, ethnicity, gender, veteran status, disability status, method of financial compensation, company type, and category. The distribution of gender in the industry is predominantly men (79.0%) who identify as Caucasian (92.8%) and with a small contingent of professionals who identify as having a disability (3.2%) or being a U.S. Veteran (13.8%). Surprisingly, there is quite a low education level for most respondents with 56% not having a bachelor’s degree or more.	Age, gender, ethnicity, disability and education
#9 [39]	The intersection of culture, disability and assistive technology	literature review	A literature review of theoretical and empirical study papers that discuss cultural aspects related to AT use or provision was performed.	The study highlights the process of cultural identity formation (also referring to disability culture) as an AT user, and on the meaning of AT to individual and family. It discusses the cultural identity of IT providers and the importance of overcoming ethnocentricity in order to be able to provide culturally appropriate AT services.	Age, gender, ethnicity, cultural identity, disability, socioeconomic status
#10 [40]	Exoskeletons for all: The interplay between exoskeletons, inclusion, gender, and intersectionality	original research	In the article, an intersectional analysis of three available commercial exos in terms of gender and diversity aspects was performed, based on a literature search and secondary empirical data acquired from manufacturers.	The study identifies features of the design of exoskeletons, which may enable or restrict device use in intersectional users. The article recommends technological solutions for the following human features, in order to ensure inclusive desigh and use: height and weight, correlated condition, capability, gender and sex, culture and inclusivity.	Body morphology (height, weight), age, socioeconomic status
#11 [41]	Implementing ethical, legal, and societal considerations in wearable robot design	theoretical article	The study is based on previous research that included expert consultations and a literature review. It applies a domain-specific approach and provides practical recommendations on how to implement ethical, legal and societal implications (ELSI) in the design, development and use of wearable robotic devices.	The authors emphasise the need of contextualising ELSI considerations in the development of wearable robots (WR), following a domain centred approach. A flowchart of action points for ELSI considerations is provided, whereby specifics of the different stages of development are summarised. These were summarised into into subjective (WR and the self: Benefits, risks and harms for Self, Body and identity impacts, the experience of vulnerability, Agency, control and responsibility), interpersonal (WR and the other: ableism and stigmatisation of the WR-supported body, Overestimation and alienation in the perception of the WR-enchanced professional body, Care-giving, dependencies and trust), and social dimensions (WR and Society: Technologisation, dehumanisation and exploitation, Social justice, resources and access, data protection and privacy, accountability and responsibility, legislation and regulation for WRs).	Disability, society

**Fig. 2:**
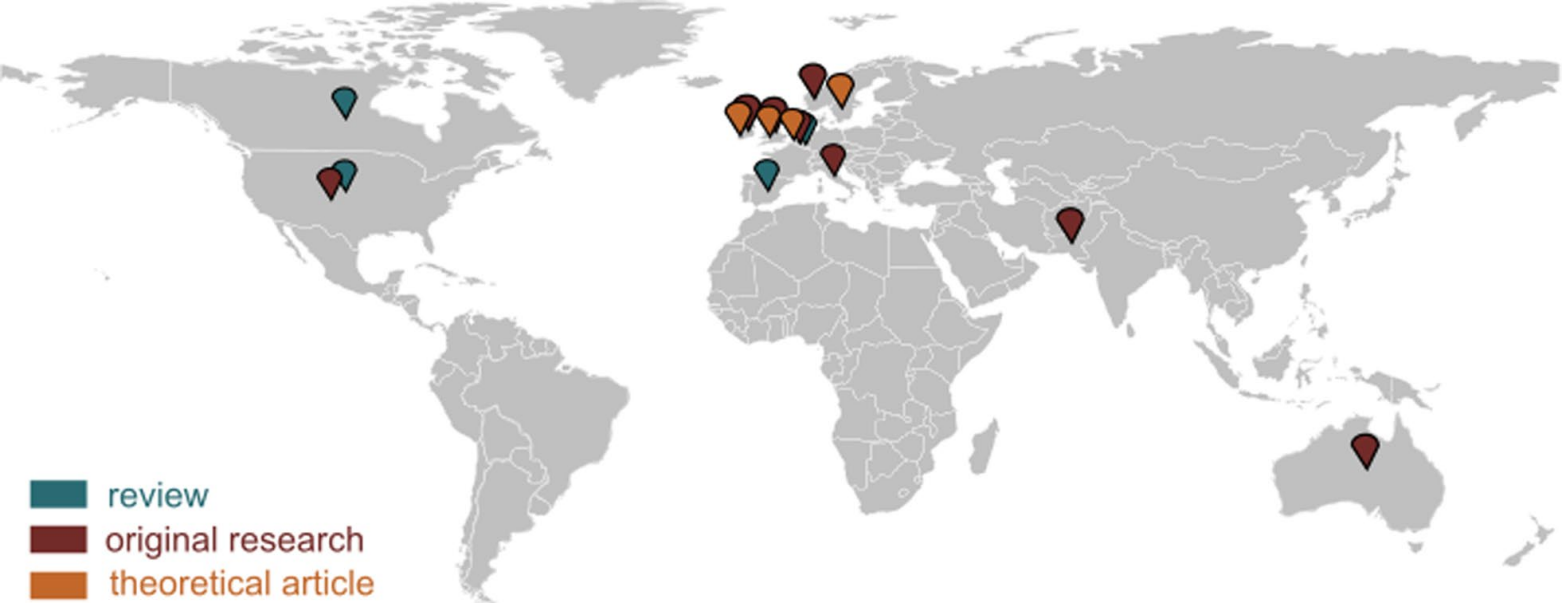
The map presents the countries where theses studies on diversity in the field of ATs were performed

As evident, there is limited research on diversity in the field of WEARTechs; eleven articles of relevance were found in the three databases that were systematically searched. Five of these extracted articles have references to a technology that can be classified as a WEARTech. Diversity and intersectionality are yet to become a consideration in the field, as hypothesised (*H1*, Table 1). At first glance, it can be observed that diversity studies in the field of WEARTechs and, in general, ATs have mainly been published from 2020 onwards, and the majority of this research took place in Europe (Fig. 2).

In 2020, Søraa and Fosch-Villaronga identified features of commercially available exoskeletons which may restrict their usage in diverse users (Article #10). Fosch-Villaronga was also one of the authors in the theoretical study by Kapeller et al (2021) which provided actionable recommendations on how to incorporate ethics and societal implications in WEARTechs R&D (Article #10). In the same year, Lusardi et al. studied lower-limb exoskeletons’ usability in every day life (Article #5). Following that, Fosch-Villaronga et al. continued working on the subject and published a review on regulatory standards for ATs in 2023 (Article #2), focusing on safety aspects, and consequently performing a study with quantitative and qualitative elements (Article #3) on the subject, focusing on lower-limb exoskeletons.

The diversity-related themes identified in the selected articles were: diverse technologies and diversity in users, use/usage and perspectives. These are summarised in the following subsections.

### Diverse technologies

**Fig. 3:**
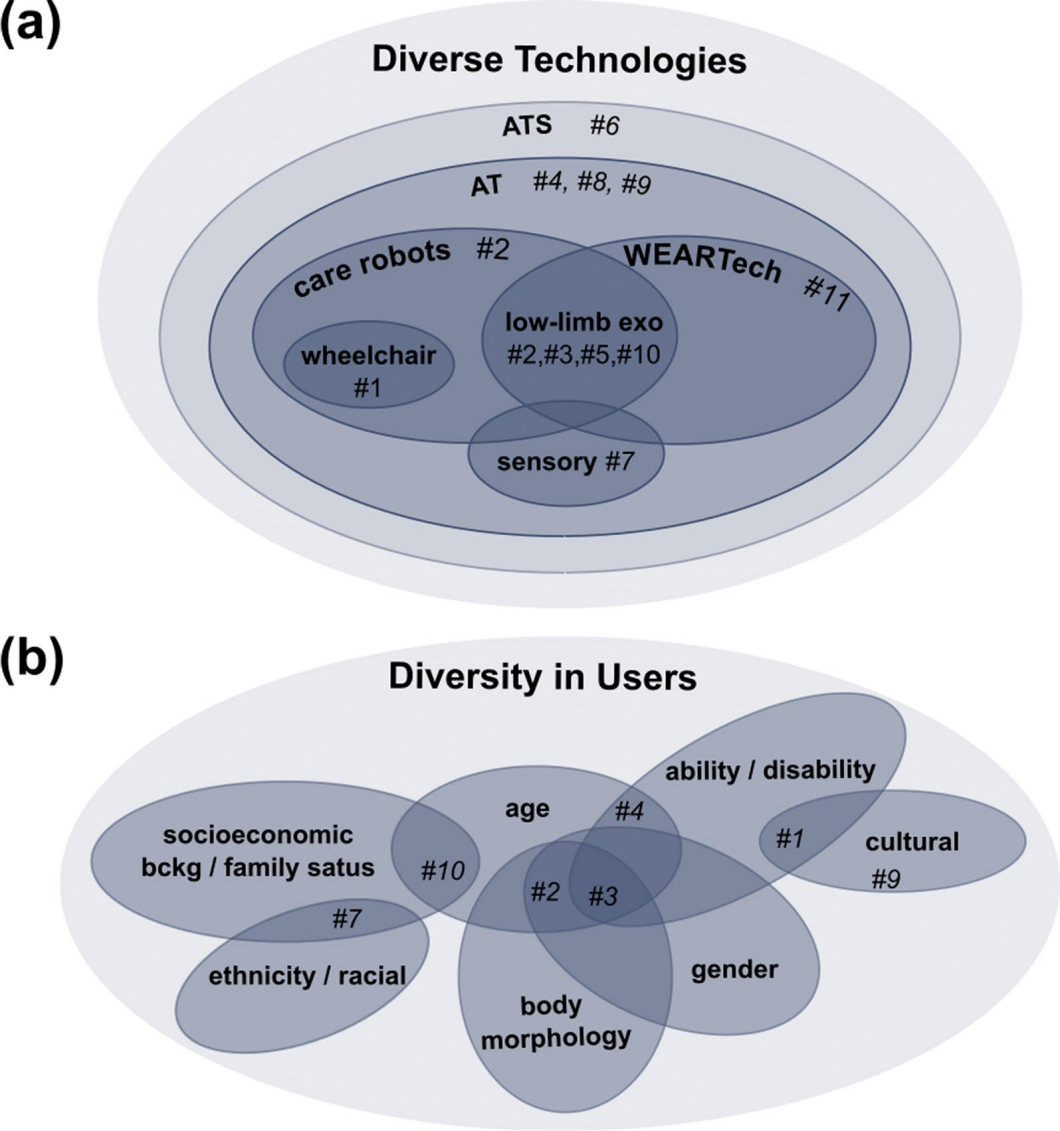
**a** The diagram presents the technologies that were addressed in the extracted literature of our systematic review. [Note: ATS=assistive technology systems, AT=assistive technology].** b** The diagram is a representation of the diversity and intersectionality in users that was identified in the systematic literature review

It is important to have a clear understanding of the technology that is at the centre of a study. Each AT can be characterised by a plethora of factors including function, purpose and usage, level of autonomy, the proximity to the human limb. Thus, diverse technologies necessitate adaptability in the requirements, evaluation techniques, and safety assessments and hazard identification approaches, for each individual system developed. Within the selected articles five different groups of ATs were identified, Fig. 3 *(a)*, among which two are wearable.

Articles #4, #8 and #9 refer to ATs but provide no clear definition for the term. The qualitative research in article #6, which focuses on the access to and assessment of ATs, considers the systems put in place for the provision, usage and assessment of ATs in addition to the product itself; the assistive technology systems (ATSs) [42]. The article takes a user-centred approach to the accessibility to exoskeletons and their assessment in order to inform the choice of technology for the individual user.

Article #2 reviews the most relevant AT regulatory standards. These standards focus on ‘personal care robots’, defined as robots that improve the user’s quality of life, which are divided into three categories; (i) the person carrier robot, (ii) the mobile servant robot—carrying objects for the human—and (iii) the physical assistant robot, that encompasses lower- and upper-limb exoskeletons (ie. wearable technologies). The authors of #2, identified gaps in the regulatory standards, ranging from the vagueness of definitions, to unaddressed safety aspects and hazards brought about by ignoring user diversity. Although, article #1 studies the “perception of ATs”, the authors represented ATs with the use of a photograph of a wheelchair. If motorised and/or sensorised, such techonology could be categorised as a person carrier robot, (i).

Three articles focus exclusively on lower-limb exoskeletons. Article #10 identifies design features of lower-limb exoskeletons that may restrict device usage in intersectional users based on literature search and secondary empirical data. The authors state concrete areas of improvement in the mechanical design of lower-limb exoskeletons. Following the review on regulatory standards (#2), the authors carry out a quantitative and qualitative evaluation of a commercial lower-limb exoskeleton in article #3. The factors identified to be responsible for the poor wearability and performance, as well as discomfort and anxiousness when using the exoskeleton, can be split into two categories: (A) the physical interface between user and exoskeleton—including attachment points, mechanical adaptability and security—and (B) the sensor interfaces and algorithms that provide situational awareness and user intent recognition. The authors of article #5 perform qualitative research on the lived experiences of using a lower-limb exoskeleton in every-day life (based on ethnographic fieldwork). An interesting, multi-dimensional facet of technologies looked at in #5 is adaptation. Adapting the device to the individual, adapting the usage of a device, adapting the way activities are performed, and acquisition of skills and re-embodiment are all components of this. Their consideration in the development of WEARTechs is important as such technologies work in close proximity to the human limbs and with certain levels of autonomy. This study also revealed that effective integration of these technologies into the life of the user requires continuous adaptation to meet the user’s needs. From our perspective, how much a user should be expected to adapt to WEARTech and to what extend WEARTech should be capable of adapting to the user is a very important question and a complex subject matter that should be addressed in the future, on a case by case basis.

Article #11 is the only study from the extracted articles that considers all wearable robotic technologies. These are wearable technologies whose operation involves dynamic human-robot interaction; which encompasses exoskeletons. The practical recommendations on the development of wearable robotics contemplate the interaction with the user, others and society and considerations on intuitiveness, maintenance, cost-effectiveness and data privacy, among others are taken into account.

Finally, article #7 performs a qualitative study on sensory technology usage that enhances every day lives of people living with impairments; these include touch sensors, fingerprint sensors and voice recognition. The article looks at accessibility, usage and their significance in individual lives. Such sensory systems are incorporated into WEARTechs and, in general, ATs.

The research so far has been skewed towards lower-limb exoskeletons and ATs that assist mobility rather than upper-limb movement, which has only started getting more popular in recent years. This is reflected in our results. There were no specific references to upper-limb technologies in our body of diversity-related literature and of the latest articles published in the WEARTechs field (extracted in *Phase II*), 37% were on the development or use of wearable upper-limb technologies and 53% on lower-limb technologies. The field of WEARTechs has not yet reached users’ needs; both therapists and patients expressed that the state of technology is not sufficiently advanced yet and still in the early days of development [43, 44]. It is thus not surprising that diversity aspects have not yet been addressed. However this omission may be part of the problem in the technology transfer from the lab to the real world.

The technologies studied in these articles range from the standard assistive tools and devices with no autonomy features, to wearable robotics; which work closely with the human body and include a level of intelligence that allows some device autonomy. The higher the autonomy level and the closer said device works to the human body the better intuitiveness and adaptability are expected from the device and it can be inferred that the more closely user and usage diversity aspects need to be considered for adequate performance.

### Diversity in users

The majority of the selected articles (7 out of 11) refer to at least one diversity characteristic of (potential) AT users. All user diversity aspects and intersectionalities found are presented in Fig. 3 *(b)*. The analysis of our body of literature showed that diversity characteristics are used to define different user groups based on one or more diversity characteristics (e.g. gender, age or ability). Some of the included articles (#2, #3) emphasise the need to consider diversity issues in the research and development of ATs and their introduction to the user.

One such sentiment is expressed in #2, the LIAISON project, which addresses the ISO standards in personal care robots which fail to address diversity aspects such as gender, sex, ability, age and how these intersect when addressing safety aspects. In article #3 the participants’ experience with a lower-limb exoskeleton and their qualitative and quantitative data demonstrated in practice the relevance of diversity factors in the various safety evaluations and standards in the device use. The following practical aspects that should be considered in relation to different users were mentioned: cognitive abilities, different learning styles, safety, different limits, mental and physical vulnerabilities, different body dimensions, interaction requirements, mental culture, physical disabilities, interaction with the robot, situation comprehension, body-reaction time and physiology (#3). The authors of article #3 conclude that neglecting diversity, equity and inclusion considerations in robot design can lead to the development of robotic systems that may compromise user safety, be discriminatory and fail to respect the fundamental rights of individuals. The reviewed articles refer to individual diversity characteristics as described in the Four-Layers of Diversity model [26]. Nevertheless, an additional diversity characteristic appeared in relation to AT, the individual body morphology.

Eight diversity characteristics were identified (within user aspects) in the literature, *age*, *ability/disability*, *gender*, *body morphology*, *ethnicity/race*, *culture*, *family status* and *socio-economic background*.

*Age* is the second most reported on participant information in the results of the *Phase II WEARTech Literature Search*; 63% of publications report on age. Within the diversity literature extracted, it is evident that *age* affects both physical aspects, like gait pattern (#3), and psychosocial aspects in the use of and adaptation to AT (#4). The two prominent meanings elderly people ascribe to AT are ‘independence’ and ‘control’ (#4). Designing ATs based on young individuals’ body morphology and gait patterns may lead to safety hazards for elderly users (#2, #3) and thus exclusion from its use (#10). The lack of a good fit of wearable ATs and lack of adaptation can lead to instability, insecurity and balance loss, causing falls, in particular in older adults (#10). This results in higher safety risks (#3). However, existing standards do not include such considerations (#2). Failure to account for *age* diversity in the potential user population will lead to the exclusion of the older user groups from using wearable ATs (#10).

The articles found do not provide much insight on *ability/disability*. The body of literature shows that similarly to elderly users, individuals with a medical condition might have more difficulties adapting to an exoskeleton and experience a higher safety risks (#3). The evidence also suggests that persons with congenital disabilities have a higher rate of successful integration of AT in their daily lives in comparison to those with other types of disabilities (#4). Furthermore, there is evidence that individuals who are familiar with the AT use have more positive perceptions of it and its usage (#1).

*Gender* considerations were found in articles which address differences in AT perceptions (#1) and those that tackle user safety and experience (#2, #3). The empirical study in article #3 reported significant differences between men and women regarding the fit and control of the exoskeleton. In spite of positive perceptions prior to the experiments, women’s experience with the device was unpleasant; it was found to be uncomfortable and felt heavier in contrast to the feedback from male participants (#3). Other studies in the field of WEARTechs have also reported on the discomfort or pain due to the position of the strapping without qualifying any diversity characteristics of the participants [4, 45, 46]. No significant differences were found between male and female participants’ perceptions of wheelchair use (#1). The *Phase II WEARTech Literature Search* indicated that *gender* is the most consistently reported on participant information aspect, with 71% of the publications recording it. A total of 370 participants’ gender was recorded and of those, 29% were female and 71% male. However, none of the studies take diversity aspects into account during the analysis of their results. This suggests that our hypotheses that participant pools lack diversity (since they are not representative of the target user group) and that there is a misrepresentation of WEARTech performance cannot be rejected (*H2* & *H3*, Table 1). If the performance of the device is different in men and women, then the results reported may be misleading. It is interesting to note that half of the studies (22/44) that reported information on gender were conducted exclusively with male participants; none of those were characterised as “male studies” and only two of them were the proof-of-concept publications. The one all-female study was described as such in the title; “in females”.

*Body morphology* emerged as an individual diversity characteristic, both due to differences in the human body based on gender, such as pelvis and chest size (#3), and due to specific body measurements, such as femur size and hip width, taken into account during the development and design of lower-limb exoskeletons (#3). Based on the results of a comparative analysis between three exoskeletons, one study identified two individual attributes related to body morphology—height and weight—that affect how a user may be included or excluded from using exoskeleton technologies (#10). Meta-data from the secondary literature search on WEARTechs (*Phase II*) indicated that 43% of the studies report on height and weight measurements, however they don’t make performance comparisons across the height and weight measurements and in the majority of cases the ranges are limited. However, neglecting differences in users’ body morphology can affect user safety and experience (#2).

In medical research there is a link between authorship genders and attention to gender and sex analysis [47]. Since gender was the one characteristic we could infer about the authorship of published articles, we tested this theory on the most recent publications in the field of WEARTechs. We found no statistically significant correlation between the two. However, most of the publications written by solely male authors, 12/18, of which only one was a proof-of-concept publication, had no female participants. Furthermore, 12/22 studies with 0 female participants had 0 female authors. It is our belief that the lack of correlation may be a result of the gender imbalance in the field, whereby the majority of WEARTech researchers are male. Taking the ‘path of least resistance’, it is more likely to perform participant studies with male individuals rather than female. In studies with $\geqslant 50\%$ women authors only 33% of the participants were female; although this is higher than the equivalent percentage for all studies (21%). Even as a female researcher, one’s vocational sphere, and maybe even social sphere, is more male dominated than that of the average individual in a society.

*Ethnicity/race* aspects were only sporadically addressed in the retrieved articles. One study showed negotiated exchanges of inclusion and exclusion, which disabled people with different racial and ethnic minority backgrounds experience when using sensory technologies (#7). Participants in the study discussed the technology’s failure to capture accents, gestures, movements, or voice commands expressed in non-normative, in this case culturally specific, manners. This is a way in which AT development could exclude people from minority or intercultural backgrounds. The study also highlighted the importance of technology education using layman terms and plain language, as complex language may be particularly challenging for those whose first language is not the local language (#7).

Only two studies from the literature found in this systematic review had some *cultural* diversity considerations (#1, #9). Article #1 compares perceptions of a wheelchair between participant groups the UK and in Pakistan. The article suggests that cultural differences in perceptions are rooted in the distinction between individualist and collectivist societies. However, it was not evident how this hypothesis is backed by the results. Article #9, points to the importance of the cultural environment of the AT user, as it will inevitably shape the perceived meaning and subsequent use of any device.

*Family status* and *socio-economic background* were also only briefly addressed by the literature but appear to be crucial factors in the use of ATs. In #7 the participants reported on the, often invisible, work of volunteers, family members and carers in adapting and using the AT, as well as enabling access through acts of interpretation and support. Device use was considered as dependant on family members’ assistance, which in turn may infringe upon privacy needs and independent living. In addition to this, financial hardship was identified as a major reason for exclusion from the use of AT (#7, #10).

### Diversity in usage

Diversity in the usage of assistive technology in individuals’ lifeworlds is another diversity aspect identified in the body of literature. Diversity in usage refers to the unique everyday experiences AT users (and their significant others) have while using specific devices, as well as to the diverse personal factors, which impact the device usability and performance. As the use of AT impacts not only the user’s lived experiences but also the way they perceive themselves and are perceived by others, the theme of identity and freedom of choice in the context of physical impairments is also relevant.

Article #5 outlines how the successful use of the exoskeleton, which requires skill acquisition and the device’s adjustment to the user and its adaptation to their lifeworld are intertwined with physical, social, cultural, environmental and psychosocial factors as well as the meaning the user attributes to mobility. The study shows how the “uniqueness of the disability” and the individual perception of disability influence whether and how individuals make use of exoskeletons. For example, while one individual rarely used their assistive device in public, barring when absolutely necessary and at important life events, another individual’s focus was on fitness, using it at tracks with family and even trying to figure out how to perform weight training with it. When considering technology, usability and adaptability, it is essential to understand what aspects of mobility matter the most to the individual in the context of their lifeworld (#5). Article #6 highlights the importance of understanding the impact of disability on people’s capabilities and functioning.

The integration of the assistive device into the personal real lifeworld also requires paying attention to the diversity of the lived experiences. Prior negative experiences with a device may lead to anxiety, activity avoidance and, thus, an increased fear of falling (#3). Furthermore, evidence suggests that individuals ascribe meanings to the assistive devices depending on factors related to: the individual’s strategy for coping with the device limitations and performance, the sense of control, the preservation of preferred self-image, the degree of importance attributed to independence, the promotion of enjoyable activity participation and the cost savings (#4). One article in particular emphasises the moral responsibility of prioritising the users’ health (#2).

Diversity in usage encompasses the concept of identity. Issues of identity have been suggested to play an important role in determining whether one decides to use the AT and in what context (#9).

Exoskeleton usage may improve the individual’s mobility and participation in everyday activities, but it also makes the individual dependent on said technology and challenges the user’s agency over their body (#2).

### Diversity in perspectives

The analysis of the selected body of literature demonstrated one further aspect of diversity in ATs; diversity in perspectives. This includes the different perspectives of relevant stakeholders (individuals who are interested and effected by the research) on the way research is being performed (#2, #3, #11), the role of  the AT and its use and how they shape the research, development, implementation and evaluation of ATs. The AT professionals are relevant for decision-making as well as accessibility to AT, as they shape the practices and narratives of technology distribution and their perspectives are addressed in article #8 and article #6. However, these are outside the scope of this study that focuses on addressing researchers and engineers in the field. Societal perspectives touch on the individual roles of AT users in society and on the paths to their exclusion or inclusion from it. An overarching aspect of diversity referred to in the selected articles is the diversity of interpretations of specific phenomena like disability, mobility or quality of life.

The selected articles refer to different approaches towards AT development. The majority of articles mention the R&D approaches they consider relevant but do not provide insights on how those are put into practice. Article #7 demonstrates how the application of a normative medicine-centred approach towards disability may affect the design of sensing technologies (#7). It shows how the medical understanding of disability was transported through to technology development, whereby “overcoming” disability through technology is considered an individual and not collective responsibility (#7). User-centred approaches were also present in found literature. Article #5 demonstrated the need for building technology with a good understanding of the user’s life, which necessitates working more closely with the users of these technologies to achieve technology acceptance. Additionally, article #9 mentions the importance of understanding the perspective of the AT user on disability. The explicit consideration of ethical, legal and societal implications (ELSI) during the development process is at the core of user-centred approaches (#11). Article #11 suggests actionable ways of translating such ELSI implications into concrete goals for developers of wearable robots. The article hypothesises that early-stage ELSI integration in the R&D process will increase users’ trust in technology which may improve device acceptability and its integration in every day life.

Another aspect of perspective diversity seen in the literature is in the appropriate hazard evaluation and safety assessment. Articles #2 and #3 demonstrate the importance of considering different perspectives, when evaluating the safety of a piece of technology: autonomy features, physical attachment points, cognitive hazards, vandalism, crowd flow and overtrust in technology. Safety and user experience are also issues that build the core of the current regulatory practice on AT. Article #11 encourages developers to apply and adapt the Guidance on Trustworthy AI [48], convened by the European Commission, to other types of data-intensive information technologies.

The societal perspectives on AT considered relevant by the included articles, on one hand, thematise the role of AT as a “bridge” and “mediator” between individual and society, facilitating the user’s participation in society (#6). On the other hand, the literature also demonstrates how negative societal perceptions of assistive devices may prevent AT users’ participation in society and lead to social exclusion (#1).

The power of interpretation as well as the dominance of certain perspectives over others is a further theme in diversity, which was found in the selected body of literature. The authors of #3 stated that more research is necessary in order to understand the role diversity considerations play in exoskeleton development and safety; including subjective, cultural and emotional aspects linked to gender stereotypes. Article #9 demonstrated how diversity in thinking and the dominance of one perspective may determine the direction of development and use of AT. This article also emphasises the culture of AT researchers, the majority of whom are university-educated, middle-class and do not consider themselves disabled (#8, #9). This position allows us to “perpetuate dominant cultural views on disability, (assistive) technology and purpose” (#9).

## Implications of research and practice

As hypothesised, there is convincing evidence in our systematic review, the literature research on WEARTechs and the background literature presented, to assert a lack of diversity in the field of WEARTech R&D; in the consideration of diversity aspects (*H1*), authorship (*H2*) and study participant pools (*H3*). The evidence supports that diversity considerations are of importance when determining the performance of these technologies, and their accessibility and introduction to the user. Although there was some evidence on how the performance of technologies is hindered by the lack of diversity considerations in the literature review, there is not enough literature on the subject to address hypothesis *H4* at the moment. We identified a significant research gap in the integration of diveristy aspects in the research methodology development and data analysis in of projects in teh field.  projects. Further quantitative and qualitative research required. There is some evidence in our literature to demonstrate that each R&D stage has unique aspects (*H5*); for example, *body morphology* emerged as a user characteristic in device performance and hazard evaluation, while aspects such as *culture* have been shown to be more significant when directly involving potential users in the process. However, again, there is not, yet, enough on the subject to determine which specific diversity aspects need to be considered at each stage for each WEARTech system.

**Fig. 4:**
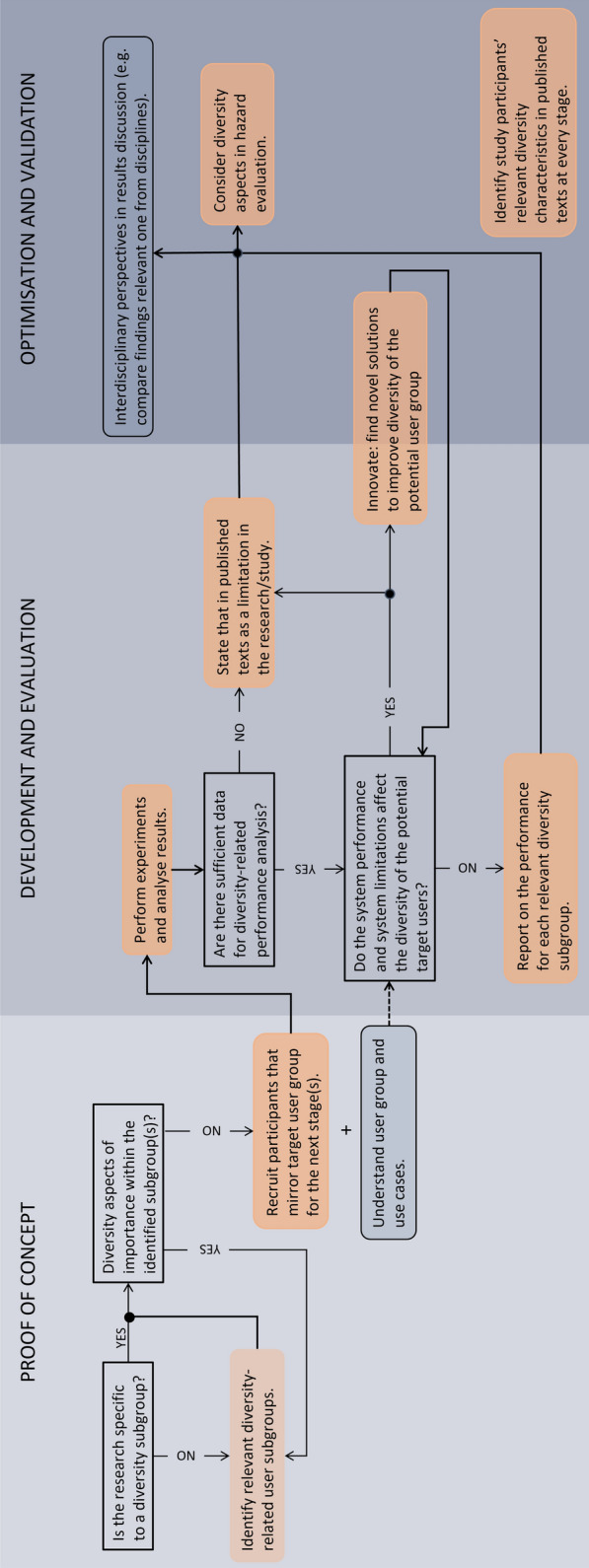
A flow diagram derived from some aspects of the recommendations (Table 5) to demonstrate a possible workflow for diversity-sensitive research practices

We transformed the findings of this literature review on diversity into practical recommendations for the WEARTech research and development. These are presented in Table 5, while a flow diagram is also provided in Appendix A as an example of how some of these can be integrated into the R&D process (Fig. 4).

In order to formulate recommendations for the integration of diversity aspects in WEARTechs R&D, conclusions from the analysis of the included literature have been combined and triangulated with existing checklists and recommendations in the field of engineering [49], design for dynamic diversity [50] and in technology and natural sciences [51]. The authors scanned and systematised the recommendations and checked their relevance to the WEARTech field. The relevant points have been adapted to our needs.

**Table 5:** Tabulation of the diversity-focused guidelines we put forward to be integrated at the different stages of the research and development of WEARTechs

Stage	Recommendations/guidelines
Funding	$\bullet$ consider funding institution’s guidelines and requirements on gender and diversity and adapt them to the project,
	$\bullet$ where feasible, involve users, their significant others and/or medical teams in the project conception and development [49],
	$\bullet$ include data on lived experiences of individuals from the intended user group in the preliminary research,
	$\bullet$ reflect on possible conflicts of interest between intended user groups or stakeholders [50],
	$\bullet$ consider project team diversity in the research and development process,
	$\bullet$ seek out exclusive funding opportunities for performing diversity-motivated research,
	$\bullet$ if feasible, initiate interdisciplinary research to minimise the risk of technology bias,
	$\bullet$ plan the recruitment of a diverse participant pool, reflective of the intended user group,
Proof of concept	$\bullet$ define the user problem/use case, investigating real-life scenarios reported in qualitative research,
	$\bullet$ report on diversity and intersectionality aspects of the study participants at all stages,
	$\bullet$ report on diversity factors in users, usage and perspectives that will be considered in future experiments,
Development & evaluation	$\bullet$ in publications, clearly state in your title/abstract when the study is gender/age/etc. specific,
	$\bullet$ recruit a diverse participant population, based on your local demographics, and consider intersectionality between the diversity factors,
	$\bullet$ incorporate gender and diversity considerations when applying for ethics approval for participant studies,
	$\bullet$ develop protocols that emulate real-world scenarios/conditions informed by use cases,
	$\bullet$ consider confidence levels among diverse device users in the processes of research, design and evaluation of WEARTechs [50],
	$\bullet$ publish data and, where feasible, report on diversity information of each participant,
	$\bullet$ perform diversity-oriented analysis on the performance and usability of the system or indicate if the data are not sufficient,
	$\bullet$ determine and report on how the technology/system limitations could affect the diversity of future users,
	$\bullet$ take advantage of the innovation potential in addressing diversity,
Validation & optimisation	$\bullet$ analyse the system’s performance considering potential user and usage diversities,
	$\bullet$ take into account diversity aspects in hazard evaluation,
	$\bullet$ where feasible incorporate interdisciplinary perspectives in publications,
Clinical study	$\bullet$ contribute towards the development of WEARTechs standards, including the technology’s introduction to diverse users,
	$\bullet$ introduce, use and promote systematic tracking of WEARTechs interventions and their outcomes in a standardised manner [52],
	$\bullet$ make individual dependability on technology explicit for the different user subgroups,
	$\bullet$ improve trust in technology, approaching intended users in a diversity-sensitive way,
	$\bullet$ consider the invisible work of others’ in the everyday use, adaptation and maintenance of the device,
	$\bullet$ clearly define the intended target group, acknowledging those who may not profit equally from the research.

The formulation of a project sets the foundation for the way the research will be performed. Our recommendations at the funding application stage are directed towards the principal investigators or other researchers involved. The European Commission (EC) and other funding institutions of research in the field have already began taking steps towards increasing awareness on inclusion and diversity. Part of the EC’s gender equality strategy, of 2020–2025, is to introduce measures to strengthen gender equality in the field of research and innovation. One such measure is requiring Marie Skłodowska-Curie Actions applicants to include considerations of gender dimension and other diversity aspects in their project’s methodology and ensure gender equality (as defined by the WHO in [53]). It should be ensured that the same values are adhered to in reports and publications resulting from said institutions' funding. Initiating diversity-oriented research may be effective in increasing the body of knowledge on the factors that could be hindering performance and optimisation of specific technologies for diverse users. In addition, it is recommended that the researchers involve the potential user group, as well as their significant others and medical teams (occupational therapists, physical therapists and doctors), during the formulation of the project. This is the stage when diversity-driven strategies should be discussed.

The minimum standard for each publication is to qualify the participant pool; ie. explicitly mention the included participants’ diversity/intersectionality aspects that are relevant. It is also crucial that we encourage data publications that report on the study’s participant diversity information available. Following the proof-of-concept stage of the system, be it an exoskeleton actuation system or a motion intent recognition algorithm, during the further development and evaluation, appropriate diversity-sensitive reporting of the results is essential. It should be discussed how the performance across diverse participant groups could vary as well as the technology’s impact on diverse potential target users. If not addressed, this could affect the usability of the technology. This is particularly important with systems that have a higher level of autonomy and take biosignals as control inputs as this could be hazardous. Most importantly, it is crucial that hazard identification is performed considering the diversity of the target user group. The evaluation of at least three of the hazards identified by article #2 in ISO 13482:2014—erroneous autonomous decisions, robot movement and shape and contact with external parts—would likely have dissimilar outcomes across different potential user groups. This reveals the essential need of developing reliable and responsible standards for WEARTechs that appropriately address all aspects of their interaction with the user and take into account diversity and intersectionality aspects. Interdisciplinary research is the key to getting the technology to the users; one needs to adapt the introduction of these ATs to diverse user groups (and use cases); taking into consideration how these users’ identities and perceptions could affect the outcome.

## Strengths and limitations

The interdisciplinary character of the study (combining engineering and socio-anthropological perspectives) enabled a multi-faceted approach towards diversity factors allowing for integrating insights from lived experiences of both technology users and researchers into the design of this article. This conceptual strength goes along with the challenge of providing tailored information for different groups of scientists in their familiar scientific jargon. We acknowledge that our recommendations may challenge the current reality of scientific projects characterised by limited durations, the need for quick turnarounds and outputs, publication-driven research, and changes in researcher teams. Nevertheless, we encourage a shift in perspective and actions towards more diversity-sensitive research and practice in order to respond to the rapidly changing environment.

Data selection, data extraction and data appraisal were performed and cross-checked by both authors allowing for internal validation of the proceedings and ongoing interdisciplinary dialog, which was crucial for the high-quality analysis. Furthermore, the search algorithm was developed based on interdisciplinary work and was refined after several test searches. Including articles on ATs instead of only focusing on WEARTechs facilitated a high degree of saturation of the relevant literature. Focusing on diversity factors in AT may have lead to misrepresentation of the field as we did not address personalised devices (sometimes with specific usage scenarios).

Addressing diversity disparities in WEARTechs is complex and cannot be solved with a single approach. This article advocates for understanding the deeper meaning of diversity for technological research rather than treating it as a checkbox. Profound changes in the field like change in demographics may enable shifting researchers’ perspectives and overall mentality. Nevertheless, it is also necessary to pay attention to the risks of diversity-oriented approaches in WEARTech development. Participants might be hesitant to join studies that collect data on various diversity factors, like body measurements or cultural background. Furthermore, achieving a diverse participant pool does not automatically solve the problem of universal applicability, since included participants cannot represent all subgroups and experiences.

Due to the limited existing research on diversity factors in WEARTech R&D, the formulated recommendations do not address all stakeholders in the process of R&D of WEARTechs equally. An example is the governmental bodies and their obligations. Although regulations and legislation are struggling to keep pace with technological advancements, it is essential to implement modernised systems that incorporate input from both scientists and users to build trust. It is important to note diversity-sensitive approaches on development, commercialisation and legislation would have to be different depending on whether the technology is new to the market, a major upgrade of an existing device or a redesign of an existing one. Another limitation of the formulated recommendations is their focus on participant studies, since existing knowledge on the subject is too limited to adequately address simulation-based research. Nevertheless, we believe that diversity factor should be incorporated in machine-based learning/simulation training. Additionally, two central aspects related to diversity-accessibility and affordability of the devices-could not be sufficiently addressed by the formulated recommendations. Although we believe that enhancing R&D practices will improve the performance and usability of WEARTechs, addressing these aspects requires action from the respective stakeholders. The same is also valid for device maintenance and repair, which were also not included in the table of recommendations. The high costs of the few highly specialised devices on the market necessitate regulation and political intervention. According to the WHO global report [1], most of the assistive products are privately developed and the greatest barrier to acquisition is affordability as most people self-fund these (which creates an even greater gap for quality of life between upper and lower economic classes).

A limitation of this study and a source of bias in our literature review is our choice of search terms in the databases chosen. Our background, experiences and influences in our respective fields influenced our choices throughout this process. An example of that is the use of more engineering-centred terms such as “exoskeleton” or “assistive technologies” while leaving out “Self-help devices”. In addition, our choice to only consider publications written in English also induces some bias in the results. It is our belief, however, that the state-of-the-art presented in international journals in this area of research is at least on par with local publications. Furthermore, our supplementary literature search only considered a small sample (100) of the newest and most relevant publications (involving participants) in the WEARTech field, a field in which there are a few hundred relevant publications per year.

To the best of our knowledge, this is the first review addressing diversity aspects in AT and the first attempt to provide practice-relevant recommendations for the R&D of WEARTechs. The limited number of available data on the role of diversity factors in AT and more specifically in WEARTechs, also lead to limitations in the validity and generalisability of the results of this systematic review. Given the limited diversity-related considerations in the AT field and the lack of evidence on technology and system performance for diverse groups, this paper aims to encourage initial steps toward including participants’ diversity characteristics in the research performed in the field. Comparing system performance based on these diversity factors will add valuable and currently missing knowledge, guiding future research in the field. It is important to note that we do not advocate for addressing all diversity factors in every study; developing technologies tailored to specific individuals for specific tasks is valid. However, it should also be noted that these individuals are most likely those with better access to ATs.

## Conclusion

Technological advances in AT in general, and WEARTechs in particular, can facilitate change in the medical and social systems, providing new opportunities for increased autonomy and participation for people with mobility impairments. In order to provide equal opportunities for all affected individuals to benefit from technological progress, the consideration of diversity should become an integral part of WEARTech R&D. This systematic review has shown that efforts in this direction are still sporadic and unsystematic. We argue that it is essential to consider diversity factors (in technology, users, usage and perspectives) and their intersectionality in the field. Applying an interdisciplinary approach, we aim to contribute to the development of a more diversity-sensitive culture in both the theory and practice of WEARTechs. Our recommendations are, what we hope to be, the first step towards more ethical and inclusive research and development of assistive and rehabilitation technologies.

## Supplementary Information

Supplementary Material 1.

Supplementary Material 2.

## Data Availability

An extended table of the data extracting during the systematic review and a table of the supplementary WEARTech literature search are available as supplementary materials.

## Appendix A: diversity-sensitive research practices

See Fig. 4

## Footnotes

1 The term ‘orthosis’ was mistakenly not included in the formulation of the search terms in February and thus a control search was performed on all databases used in May 27th. This produced the same number of results and no additional relevant articles.
